# Optimal Packaging of FIV Genomic RNA Depends upon a Conserved Long-range Interaction and a Palindromic Sequence within *gag*

**DOI:** 10.1016/j.jmb.2010.08.019

**Published:** 2010-10-15

**Authors:** Tahir A. Rizvi, Julia C. Kenyon, Jahabar Ali, Suriya J. Aktar, Pretty S. Phillip, Akela Ghazawi, Farah Mustafa, Andrew M.L. Lever

**Affiliations:** 1 Departments of Microbiology & Immunology, Faculty of Medicine and Health Sciences (FMHS), United Arab Emirates University (UAEU), Al Ain, UAE; 2 Department of Medicine, Addenbrooke’s Hospital, University of Cambridge, Cambridge CB2 2QQ, UK; 3 Department of Biochemistry, Faculty of Medicine and Health Sciences (FMHS), United Arab Emirates University (UAEU), Al Ain, UAE

**Keywords:** FIV, feline immunodeficiency virus, HIV, human immunodeficiency virus, SIV, simian immunodeficiency virus, UTR, untranslated leader region, mSD, major splice donor, MPMV, Mason-Pfizer monkey virus, pal, palindromic, DIS, dimerization initiation site, RT-PCR, reverse transcriptase, LTR, long terminal repeat, DIS, dimerization initiation site, RPE, relative packaging efficiency, feline immunodeficiency virus (FIV), retroviral RNA packaging, long-range interaction, palindromic sequences (pal), lentiviral vectors and gene therapy

## Abstract

The feline immunodeficiency virus (FIV) is a lentivirus that is related to human immunodeficiency virus (HIV), causing a similar pathology in cats. It is a potential small animal model for AIDS and the FIV-based vectors are also being pursued for human gene therapy. Previous studies have mapped the FIV packaging signal (ψ) to two or more discontinuous regions within the 5′ 511 nt of the genomic RNA and structural analyses have determined its secondary structure. The 5′ and 3′ sequences within ψ region interact through extensive long-range interactions (LRIs), including a conserved heptanucleotide interaction between R/U5 and *gag*. Other secondary structural elements identified include a conserved 150 nt stem–loop (SL2) and a small palindromic stem–loop within *gag* open reading frame that might act as a viral dimerization initiation site. We have performed extensive mutational analysis of these sequences and structures and ascertained their importance in FIV packaging using a *trans*-complementation assay. Disrupting the conserved heptanucleotide LRI to prevent base pairing between R/U5 and *gag* reduced packaging by 2.8–5.5 fold. Restoration of pairing using an alternative, non-wild type (wt) LRI sequence restored RNA packaging and propagation to wt levels, suggesting that it is the structure of the LRI, rather than its sequence, that is important for FIV packaging. Disrupting the palindrome within *gag* reduced packaging by 1.5–3-fold, but substitution with a different palindromic sequence did not restore packaging completely, suggesting that the sequence of this region as well as its palindromic nature is important. Mutation of individual regions of SL2 did not have a pronounced effect on FIV packaging, suggesting that either it is the structure of SL2 as a whole that is necessary for optimal packaging, or that there is redundancy within this structure. The mutational analysis presented here has further validated the previously predicted RNA secondary structure of FIV ψ.

## Introduction

The feline immunodeficiency virus (FIV) is a lentivirus that causes a prolonged disease in domestic cats that is similar in characteristics to AIDS in humans caused by the human immunodeficiency virus (HIV).^1,2^ Because of many similarities between FIV and HIV, FIV is an important laboratory model to study HIV infection and pathogenesis and for the development of HIV vaccines and therapies.^3–5^ In addition, being a lentivirus with a unique ability to infect and transduce non-dividing cells and due to its greater evolutionary distance from primate retroviruses, FIV is considered a potential vector system for human gene therapy.^6–10^ However, before FIV based vectors can be exploited for human gene therapy, it is crucial that the relevant aspects of FIV replication are understood.

Efficient and specific packaging or encapsidation of retroviral RNA is one of the essential steps in retroviral replication during which two copies of “full-length” unspliced genomic RNA are encapsidated preferentially into the assembling virus particles from a large milieu of cellular and viral RNAs in the host cell cytoplasm. The process of specific encapsidation involves the recognition of particular sequence(s) and/or structural element(s) of the genomic RNA located at its 5′ end termed the “packaging signal” (ψ).^11–18^ In addition, for at least some retroviruses, the 5′ end of U5, the 5′ end of the *gag* gene, and a region near the 3′ end of viral RNA also facilitate packaging in conjunction with the untranslated leader region (UTR).^17,18^ In general, simple retroviruses are thought to contain a more discrete ψ localized to small segments at the 5′ end of the viral genome,^12,13^ while those of complex retroviruses, like FIV, are thought to be more spread out.^19^^–^^24^ The emerging picture of retroviral genomic RNA packaging suggests that the 5′ end of the retroviral genome contains the major packaging determinants of the virus but the nuances of the packaging signals of each retrovirus must be determined empirically.^18^ The specific capture of the ψ containing genomic RNA by the assembling virus requires the interaction of ψ with the zinc finger domains of the nucleocapsid region of the Gag polyproteins.^14,15,17,18^ Furthermore, because the specificity of packaging can be exchanged in some retroviruses by the substitution of ψ that have no sequence homology,^25^ the process of encapsidation is likely to involve recognition at the structural level. RNA secondary structure prediction analyses have identified stem–loop structures as part of the packaging signals for many simple and complex retroviruses.^17^^,^^18^

Due to the increasing interest in FIV-based vectors for human gene therapy, FIV RNA packaging has been investigated extensively in the recent years. A series of studies have suggested that similar to the packaging determinants of the primate lentiviruses, HIV and simian immunodeficiency virus (SIV), the packaging determinants of FIV are complex and multipartite; however, the precise location and the relative contribution of each of these determinants remain somewhat debatable.^19,20,22^^–^^24^ Using a systematic deletion analysis of the FIV 5′ UTR and *gag* sequences of subgenomic constructs, it has been shown that the FIV packaging determinants consist of two discontinuous core regions. One located upstream of the major splice donor (mSD) from R/U5 at the 5′ end to the first 150 bp of UTR, while the other is found within the first 100 nt of the *gag* gene, both of which are equally important and required simultaneously for packaging.^19,20,22,23^ The intervening sequences between these two discontinuous regions are dispensable without affecting either RNA packaging or propagation.^24^ In addition, it has been shown that sequences within the FIV 3′ long terminal repeat (LTR) also contain minor packaging determinants but these sequences contribute to a lesser extent.^21^

**Fig. 1:**
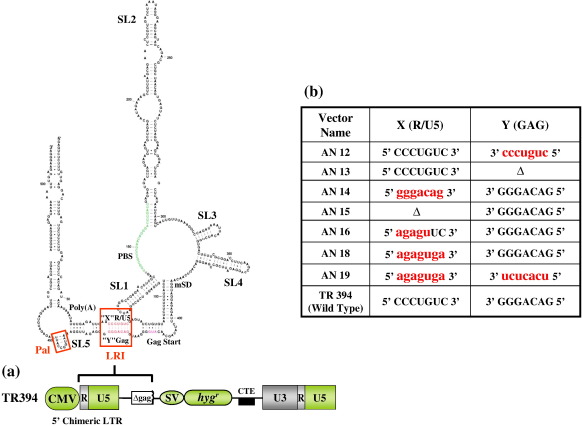
(a) A representation of TR394, the wild type FIV-based transfer vector in which the region between R and Gag that has been shown to be involved in RNA packaging is depicted by a bold line. The same region folds into the illustrated RNA secondary structure that was used to introduce mutations.^31^ The complementary heptanucleotides involved in LRI are boxed. (b) A table outlining the deletions and/or substitutions in the complementary heptanucleotides at both locations of LRI (R/U5 “X” and Gag “Y”) in order to lose complementarity or to re-establish artificial complementarity with heterologous sequences. Nucleotides shown in lower case represent the mutations introduced in the wild type heptanucleotides. Pal, palindromic sequence; LRI, long-range interaction; poly(A), polyadenylation sequence; PBS, primer-binding site; mSD, major splice donor; SV, simian virus 40 promoter/enhancer; *hyg*^*r*^, *hygromycin resistance* gene; CTE, constitutive transport element.

Similar to other retroviruses, such as HIV, SIV and Mason-Pfizer monkey virus (MPMV),^18,26–30^ the FIV packaging signal RNA, composed of the 5′ 511 nt of the FIV genomic RNA, folds into several stem–loop structures shown in Fig. 1a.^31^ Using minimal free-energy structural predictions, biochemical probing, and phylogenetic analyses, we demonstrated five conserved stem–loops (SL1–SL5) and a conserved long-range interaction (LRI) between complementary heptanucleotides in R/U5 (nt 289 5′ CCCUGUC 3′ nt 295) and *gag* (nt 644 3′ GGGACAG 5′ nt 638). In addition to the LRI, we identified a prominent 10 bp (nt 657 5′ AAUGGCCAUU 3′ nt 666) 100% palindromic (pal) sequence in stem–loop 5 within the Matrix coding region of *gag*.^31^ In sharp contrast to this work, James and Sargueil proposed an alternative secondary structure for FIV RNA in which the LRI was not observed.^32^ Furthermore, they did not identify the conserved pal sequence within *gag* proposed by Kenyon *et al*.^31^ but instead highlighted a much less conserved pal sequence in the UTR. One possible reason for their alternative structure and lack of identification of the conserved pal in *gag* was their use of multiple sequences derived from the same molecular clone to support their analysis. We have recently performed further structural and functional analysis of the FIV leader in which we have confirmed that the proposed dimerization initiation site (DIS) is in a double-stranded conformation consistent with a loop–loop dimerization initiation reaction and that mutants that disrupt the palindrome abrogate this paired structure (data not shown).

LRIs involving complementary sequences have been shown to exist in other lentiviruses^33^^–^^36^ and disruptive mutations of these affect important steps in the viral life-cycle, including RNA packaging and dimerization.^34,35^ The significance of palindromic sequences in lentiviral replication is also well established as dimer linkage sites^37^ and was affirmed recently in the case of HIV-2 as affecting both RNA packaging and dimerization.^38^^–^^40^

The RNA secondary structure of the FIV leader sequence we proposed earlier has not been tested genetically although it provides a mechanistic explanation for the bipartite ψ established by mutagenesis.^31^ Therefore, to establish the biological significance of different structural components of the proposed structure and to provide functional evidence for the existence of the LRI and the role of pal in FIV RNA packaging, we introduced a series of mutations including deletions/substitutions and compensatory mutations in the proposed structure. These mutations were tested in a biologically relevant *in vivo* packaging and transduction assay to determine their effects on packaging and replication efficiency of FIV transfer vectors.^41^

## Results

### Three plasmids *in vivo* packaging and transduction assay to measure FIV RNA packaging and propagation efficiencies

The study of RNA packaging in FIV is limited because of the essential nature of the 5′ *gag* sequence as a component of the packaging signal. This makes examination of packaging using full-length wild type virus highly problematic because mutations will affect the *gag* open reading frame. Point mutations altering sequence whilst maintaining coding are a possibility but such mutants often have unexpected *cis* effects such as on RNA trafficking.^42^ For this reason, we performed the assays using the three plasmid co-transfection assay for FIV described earlier.^41^ Briefly, the assay requires co-transfection of a transfer vector (containing a marker gene), a packaging construct (MB22) and an envelope expression plasmid (MD.G). Such a strategy results in the generation of virus particles containing the packaged RNA, the replication of which is limited to a single round. These virus particles can be used to (1) examine the packaging efficiency directly by measuring their viral RNA content using reverse transcriptase PCR (RT-PCR) and (2) infect target cells resulting in the transduction of these cells with the marker *hygromycin resistance* gene thus allowing monitoring of vector RNA propagation. The number of hygromycin-resistant (Hyg^r^) colonies obtained should correlate with the viral RNA content, giving us an indirect measurement of transfer vector RNA packaging efficiency. This *trans*-complementation assay allowed us to mutate the RNA secondary structure of the sequences that have been implicated in RNA packaging in the sub-genomic viral context without affecting the overlapping Gag/Pol reading frames because these gene products were provided *in trans* from a separate plasmid.

### Validation and biological significance of R/U5-Gag long-range interaction (LRI)

Earlier, we showed that the first 105 nucleotides of FIV genomic RNA are involved in extensive LRIs with those at the 3′ end of mSD, including the first 100 bp of Gag, which includes a conserved complementary heptanucleotide interaction between R/U5 (5′ CCCUGUC 3′) and Gag (3′ GGGACAG 5′).^31^ Other regions beside the complementary heptanucleotides involved in LRIs could possibly be involved in RNA packaging; however, the region at the 5′ end is composed largely of A-U and G-U wobble pairs and similarly the 3′ end flank is G-C poor; therefore, the metastability of these regions might have a role in facilitating structural changes, as has been suggested earlier for other viral systems.^43^ However, for this study, we focused on the complementary heptanucleotide interaction and not the flanking regions showing widespread LRI because it is only the heptanucleotide LRI itself that is conserved between strains of FIV that infect the other feline species (e.g. cougars, lions, Pallas cats), as well as amongst domestic cat FIV strains.^31^ Therefore, in an attempt to characterize the biological significance of the LRI involving conserved complementary heptanucleotides, mutations were introduced in the heptanucleotides as shown in Fig. 1b and tested in the three plasmids *trans*-complementation assay.

Briefly, the wild type (TR394) and mutant transfer vectors were co-transfected in 293T producer cells along with MB22 and MD.G in the presence of a control plasmid, pGL3, expressing firefly luciferase. At 72 h after transfection, supernatants containing virus particles were harvested and used to infect HeLa CD4^+^ cells as well as to isolate packaged virion RNA. Some of the transfected cells were used to prepare whole cell protein extracts to determine the transfection efficiencies and the rest were fractionated into nuclear and cytoplasmic RNA fractions. The infected HeLa CD4^+^ cells were selected with medium containing hygromycin B to monitor the propagation of the transfer vector RNAs.

**Fig. 2:**
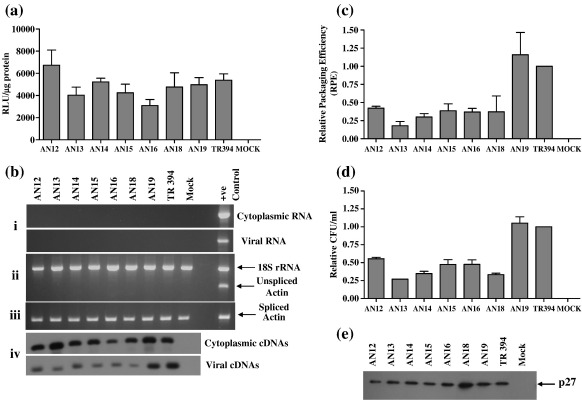
Role of the complementary heptanucleotides in R/U5 and Gag involved in LRI towards FIV RNA packaging and propagation. (a) Transfection efficiency of mutants and wild type transfer vector as assessed by the firefly luciferase activity from the co-transfected pGL3 control DNA using the dual luciferase assay kit. RLU, relative light units. The average of the data from three independent representative experiments is shown. (b) RT-PCR of viral and cytoplasmic RNA fractions with appropriate controls. (i) Amplification of the DNase-treated cytoplasmic (upper panel) and viral (lower panel) RNA preparations using transfer vector specific primers. (ii) and (iii) Controls for the nucleocytoplasmic fractionation technique; (ii) amplification of unspliced β-actin mRNA and 18S rRNA; (iii) amplification of spliced β-actin mRNA. (iv) Representative Southern blot of the amplified products following RT-PCR that was conducted on transfer vector cytoplasmic cDNAs (upper panel) and viral cDNAs (lower panel) using vector-specific primers. (c) Relative packaging efficiency (RPE) of transfer vector RNAs. As described in Materials and Methods, the amount of genomic RNA packaged for each mutant was compared with the wild type (TR394) after quantification of the bands obtained following semi-quantitative RT-PCR. (d) Relative hygromycin-resistant (Hyg^r^) colony-forming unit (CFU)/ml for mutant transfer vectors reflecting the relative RNA propagation efficiencies. The CFU/ml value expressed for each mutant is relative to the wild type (TR394) and data were derived after normalization to the transfection efficiencies. (e) Representative western blot conducted on the pelleted viral particles. The RPE and relative CFU data represent the mean of at least three independent transfection and infection experiments testing all mutants except for the relative CFU/ml value of AN13. RLU, relative light units/μg of protein.

To determine that transfer vector RNAs were expressed stably and subsequently packaged into the budding virions, both cytoplasmic and viral RNA preparations were analyzed by RT-PCR. However, to eliminate the possibility of the presence of any contaminating plasmid DNA in the RNA preparations before RT-PCR, RNAs were treated with DNase and amplified. The absence of any detectable level of plasmid DNA contamination was confirmed by the lack of amplification using vector-specific primers in both cytoplasmic (Fig. 2b.i, upper panel) and viral RNA preparations (Fig. 2b.i, lower panel) following 30 cycles of PCR. Having confirmed this, RNA preparations were reverse transcribed to make the cDNA.

To confirm that transfer vector RNAs were properly transported to the cytoplasm, we ensured that there was no artifact in our fractionation technique, which might have compromised the nuclear membrane integrity resulting in the leakage of the transcribed RNAs to the cytoplasm. Towards this end, the cytoplasmic RNA fractions were tested by RT-PCR to ensure the absence of any amplifiable unspliced β-actin mRNA signal because this is found exclusively in the nucleus.^44^ Lack of any detectable amplification of unspliced β-actin message in the cytoplasmic fractions (Fig. 2b.ii), although it could be detected in the nuclear fractions (Fig. 2b.ii, last lane), suggested that our cytoplasmic RNA preparations were *bona fide*. The presence of spliced β-actin mRNA (Fig. 2b.iii) in the cellular fraction containing the transfer vector RNA confirmed that the vector RNA had been transported to the cytoplasm. Our interpretation was based on the absence of unspliced β-actin mRNA in the cytoplasmic fraction; therefore, to ensure that each cytoplasmic sample in the unspliced β-actin PCRs contained amplifiable cDNAs, PCRs were conducted in the presence of primers/competimers for 18S ribosomal RNAs as an ancillary control. Amplification of 18S ribosomal RNA in the multiplex PCR (30 cycles) verified the presence of amplifiable cDNA (Fig. 2b.ii). Next, the cytoplasmic cDNAs were amplified for 15, 20 and 25 cycles using vector-specific primers and were found to be expressed stably (Fig. 2b.iv, upper panel). Finally, viral cDNAs were amplified using the same set of primers (as for the cytoplasmic cDNAs) for the same number of cycles of PCR to analyze the relative packaging efficiency (RPE) of each mutant transfer vector RNA (Fig. 2b.iv, lower panel). Amplified products were processed for Southern blot analysis and the DNA for probe was generated by PCR using the same vector-specific primers that were used to amplify the cytoplasmic and viral cDNAs.

As shown in Fig. 1b, the mutations in the complementary heptanucleotides were designed to disrupt the complementarity and therefore the LRI. Semi-quantitative RT-PCR results from a minimum of three independent experiments were quantified to determine the RPE for each LRI mutant, which was measured by calculating the ratio of packaged mutant RNA to wild type RNA relative to the ratio of the two RNAs in the cytoplasm as described in Materials and Methods. These analyses revealed that the mutations that were introduced to disrupt the LRI between the heptanucleotides affected transfer vector RNA packaging efficiency significantly when compared to the wild type transfer vector TR394, with a reduction in RPEs in the range 2.4–5.6-fold (*P* *=* 0.004–0.1; Fig. 2c). In concordance with the results for RNA packaging, we observed that mutants in which RNA packaging was compromised showed equally severe defects in RNA propagation; i.e. the appearance of Hyg^r^ colonies in the infected cultures was found to be significantly low compared to the wild type (Fig. 2d). This was despite the fact that the transfection efficiencies were within twofold for repeated experiments (Fig. 2a) and semi-quantitative RT-PCR analysis of all mutants revealed steady-state levels of expression of cytoplasmic RNA relative to the wild type (TR394) (Fig. 2b.iv; upper panel); therefore, such a low level of Hyg^r^ colonies in the infected cultures could be attributed to the reduced mutant RNA packaging. Furthermore, on the basis of transfection efficiency (when normalized supernatant volumes from the transfected cultures were pelleted to isolate packaged viral RNA), similar amounts of virions were used to isolate viral RNA for the mutants and the wild type transfer vectors as shown by western blotting (Fig. 2e). On the basis of the mutational analysis presented in this section, our data suggested that there is a biologically significant LRI between these two complementary heptanucleotides and any perturbation in these sequences that results in the loss of complementarity negatively affected RNA packaging.

To ascertain whether it is the primary sequence of the complementary heptanucleotides or whether heterologous heptanucleotides maintaining the complementarity between these locations (R/U5 and Gag) would be sufficient to maintain LRI and therefore RNA packaging, we created two mutant clones (AN18 and AN19) to address this paradigm. In the case of AN18, heptanucleotide sequence “X” (5′ CCCUGUC 3′) in R/U5 was substituted with heterologous sequence of equal length (5′ agaguga 3′) such that it would lose its complementarity with the sequence “Y” (3′ GGGACAG 5′) in Gag (Fig. 1b). As shown in Fig. 2c, this destabilizing substitution mutation in AN18 diminished FIV RNA packaging efficiency by 2.7-fold compared to the wild type TR394. The reduced FIV RNA packaging of AN18 correlated well with the vector RNA propagation results and showed a threefold reduction (*P* = 0.001) in transduction efficiency (Fig. 2d). However, in a compensatory mutation approach, the negative effects on RNA packaging and propagation were restored to the wild type levels when, in AN19, native heptanucleotide sequence “Y” (3′ GGGACAG 5′) in Gag was substituted with a heterologous sequence (3′ ucucacu 5′) that was complementary to the heterologous heptanucleotide sequence “X” (5′ agaguga 3′) in R/U5 in the case of AN18 (Fig. 2c and d).

**Fig. 3:**
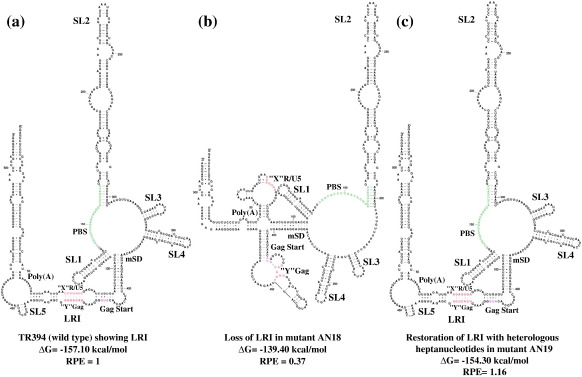
A possible model of the structural change caused by mutations in complementary heptanucleotides based on free energy minimization alone using the Mfold program with biochemical constraints as described earlier.^31^ This is not suggested to be a definitive structural model but is intended to illustrate how the mutation might interrupt the folding caused by the intact heptanucleotide long-range interaction. (a) Proposed structure of the wild type (TR394) showing LRI between complementary heptanucleotides.^31^ (b) Folding pattern of AN18 containing heterologous heptanucleotides in R/U5 “X” resulting in the disruption of the predicted RNA secondary structure. (c) Folding prediction of AN19 containing heterologous heptanucleotide sequence in Gag “Y” complementary to the heterologous heptanucleotide sequence introduced in AN18 (R/U5 “X”), resulting in the restoration of both the overall RNA secondary structure and RNA packaging to the wild type (TR394) level. Pal, palindromic sequence; LRI, long-range interaction; poly(A), polyadenylation sequence; PBS, primer-binding site; mSD, major splice donor; RPE, relative packaging efficiency.

The loss of LRI in AN18 creates a disrupted predicted secondary structure by Mfold analysis compared to the wild type, with either a change of register between the two sequences involved in the LRI or the stabilization of separate R/U5 and *gag* stem–loops, although stem–loops 1–4 remain intact (Fig. 3b), and a less favorable free energy (Δ*G* = – 139.4 kcal/mol *versus* – 157.1 kcal/mol for the wild type). In AN19, the LRI was restored with the substitution of heterologous heptanucleotides, and Mfold analysis predicted the restoration of wild type secondary structure, with a similar free energy (Δ*G* = – 154.3 kcal/mol; Fig. 3c). These results further demonstrated that it is the complementarity between the heptanucleotides establishing LRI that is crucial for RNA packaging rather than the primary sequence itself, because re-establishing the complementarity using heterologous heptanucleotides restored packaging to the wild type levels.

### The role of a conserved 10 nt palindromic sequence in the core packaging determinant of FIV in RNA packaging

**Fig. 4:**
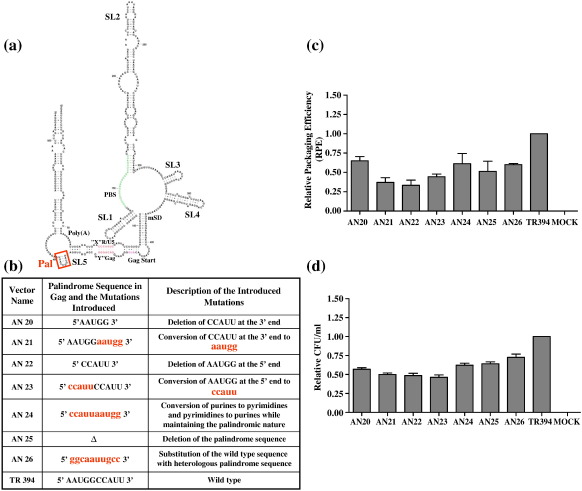
Effects of mutations in the pal sequence in Gag on FIV RNA packaging and propagation. (a) RNA secondary structure of the FIV 5′ RNA genome described earlier.^31^ The 10 nt pal sequence in which the mutations were introduced is boxed. (b) A table describing the mutations introduced into pal in order to lose its palindromic nature and/or substitution of the wild type pal with a heterologous pal sequence. Nucleotides shown in lower case represent the mutations introduced in the wild type pal. (c) RPE of transfer vector RNAs containing mutations in pal as described in Materials and Methods and in Fig. 2c. (d) Relative propagation efficiency of the transfer vector RNA containing mutations in pal expressed as Hyg^r^ CFU/ml of viral supernatant that was used to infect target cells and the data were derived after normalization to the transfection efficiency. The RPE and the propagation data represent the mean of a minimum of three independent experiments. Pal, palindromic sequence; LRI, long-range interaction; poly(A), polyadenylation sequence; PBS, primer-binding site; mSD, major splice donor.

Earlier, we identified a conserved prominent pal sequence (5′ AAUGGCCAUU 3′) with 100% autocomplementarity in a stem–loop within the first 100 bp of the *gag* open reading frame (Fig. 4a).^31^ This region of the FIV genome is needed for RNA packaging,^19-24^ advocating a potential role in packaging for this stem–loop at the structural level. Because pal-mediated RNA dimerization has been shown to play an important role in the packaging and replication in other lentiviruses,^39,40,45^ we wanted to evaluate the functional importance of this pal sequence. In order to do so, two sets of mutants were created, which included deleting or scrambling the pal sequence to lose its palindromic nature or replacing it with a heterologous sequence of identical length with autocomplementarity (Fig. 4b). Analysis of multiple experiments showed that the mutations in pal did not affect the steady-state level of cytoplasmic RNA and that nearly equal amounts of particles were produced in the transfected cultures for both the mutants and the wild type transfer vectors (data not shown). Results of a minimum of three independent experiments after normalization to transfection efficiency were computed to calculate the RPE of each mutant transfer vector RNA. As shown in Fig. 4c, pal mutants exhibited various degrees of reduction in RPEs (1.5–3 fold; *P* *=* 0.004–0.02) compared to the wild type and the RPE data corroborated well with the propagation data (Fig. 4d) as measured by counting the number of Hyg^r^ colonies in the infected cultures.

The first set of mutants (AN20–AN23; Fig. 4b) contained either deletion or duplication of the first or the second half of pal. AN20, which contains a 5 bp deletion at the 3′ (ΔCCAUU), reduced the RPE by 1.5-fold and RNA propagation by 1.8-fold when compared to the wild type (Fig. 4c and d). AN22, which contains a 5 bp deletion at the 5′ (ΔAAUGG; Fig. 4b) showed a somewhat more pronounced effect, a threefold decrease in RPE and a 2.1-fold decrease in vector RNA propagation compared to the wild type (Fig. 4c and d), suggesting that the 5′ sequence of pal might be more important for packaging than the 3′ half, although the lengths of the deleted sequences were identical. To exclude the possibility that the reduced length of the sequence was responsible for the defect in FIV replication, AN21 and AN23 mutant clones were created, which contained duplication of the 5′ (first half) and the 3′ (second half) of pal respectively (Fig. 4b). The RPE of AN21 and AN23 was found to be lowered by 2.7 (*P* *=* 0.009) and 2.2 (*P* *=* 0.004) fold, respectively, compared to the wild type, whereas propagation was lowered by twofold and 2.2-fold, respectively (Fig. 4c and d), thereby excluding such an effect of reduced sequence length. In a more drastic mutation, AN25, we deleted the entire pal, which resulted in nearly twofold drop in RPE compared to wild type, TR394 (Fig. 4c).

In order to determine whether it is the primary sequence or the palindromic nature that is important for FIV RNA packaging, we created two additional mutants (AN24 and AN26; Fig. 4b). In the case of AN24, purines were replaced with pyrimidines and *vice versa* therefore maintaining the palindromic nature of the sequence. In the case of AN26, the wild type pal was replaced with a heterologous pal sequence of equal length. Testing these mutants (AN24 and AN26) showed that both RPE and propagation were reduced; RPE was reduced by 1.6-fold for both mutants, whereas the propagation was reduced by 1.6-fold and 1.4-fold, respectively, compared to the wild type (Fig. 4c and d). These results suggested that the primary sequence of pal as well as its palindromic nature is important because disruption of the palindromic nature or its substitution with the heterologous pal of identical length affected both RNA packaging and propagation negatively.

Most of the mutants tested had packaging defective phenotype; however, structure predictions did not show drastic changes in the overall structure (data not shown), further arguing in favor of the primary sequence being more important than its structure for RNA packaging. One caveat is that structural predictions are based on a monomeric RNA and it is not possible to predict exactly how the mutations might affect the intermolecular interaction during dimerization, which also influences packaging in all other retroviruses studied so far.

### Mutational analysis of stem–loop 2 (SL2)

**Fig. 5:**
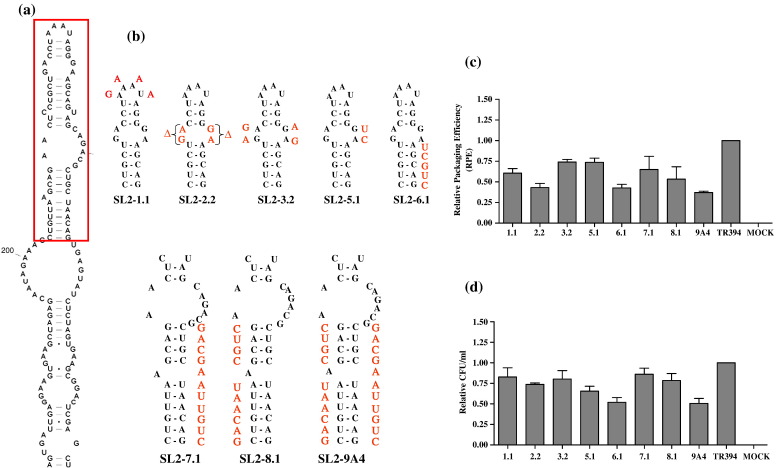
Analysis of the deletion/substitution mutations introduced into the apical region of SL2. (a) Part of the RNA secondary structure depicting SL2 with the region in which mutations were introduced is highlighted by an unbroken-line box. (b) Nature of the deletion/substitution and destabilization/compensatory mutations that were introduced into the upper region of SL2. The precise nature of each mutation is shown separately along with the adjoining area of the RNA secondary structure. (c) RPEs of transfer vector RNAs containing mutations in the upper region of SL2. (d) Relative transfer vector RNA propagation for each mutant expressed as Hyg^r^ CFU/ml of the viral supernatant. The RPE and propagation data are derived from at least three independent transfection and infection experiments after normalization to the transfection efficiency.

Our earlier structure analysis and biochemical probing of the 5′ end of FIV RNA genome showed a long (approximately 150 nt) and very stable stem–loop (SL2), which includes a region that has been implicated in FIV RNA packaging.^19,24^ To ascertain the importance of SL2 for RNA packaging, we introduced a series of substitution, deletion and compensatory mutations. The rationale behind introducing these mutations was based on the fact that if a particular component of SL2 is important for packaging at the structural level, mutations in this region should disrupt the intramolecular base pairing and should in turn affect RNA packaging. On the other hand, the introduction of compensatory mutations should recreate the stem–loop, restoring its structure and function, which in turn should restore RNA packaging. We first introduced mutations in the uppermost part of SL2, which are shown in Fig. 5a and b.

Semiquantitative RT-PCR was conducted on both the viral and cytoplasmic RNAs to calculate the RPE and the data obtained from a minimum of three independent experiments are shown in Fig. 5c and d. Substitution of the apical loop (AAAU to GAAA) in SL2-1.1 affected both RNA packaging and propagation modestly with a reduction of 1.6-fold (*P* = 0.01) in RPE and 1.2-fold in propagation when compared to the wild type (Fig. 5c and d). Substitution of either the flanking four or two purines in the bulge in the case of SL2-3.2 and SL2-5.1 mutants, respectively, showed a 1.4-fold reduction in RPE; however, the deletion of these four flanking purines in the bulge (SL2-2.2) reduced the RPE by more than twofold (*P* = 0.008; Fig. 5c). This was despite the fact that the transfection efficiencies for the repeated experiments were well within twofold (data not shown), the intracellular transfer vector RNAs for each of the mutants were expressed efficiently, and similar amounts of virions were used to isolate packaged RNA as determined by western blot (data not shown). These results suggest that the native purine bases on both sides of the bulge might be involved in the recognition of the RNA for packaging because substitution with the purines in the opposite orientation (SL2-3.2), or with pyrimidines “UC” on only one side (SL2-5.1) did not exert significant packaging or propagation defects; whereas the loss of the bulge (SL2-2.2) reduced RPE by 2.3-fold. Alternatively, as shown in other retroviruses, the ability of the RNA to unwind during packaging might be critical and the bulge-closing mutations would be predicted to stabilize the terminal loop, thus reducing the metastability of the overall structure.^18^

Next, to disrupt the intramolecular base pairing in the stem just below the bulge in the case of SL2-6.1 (Fig. 5a and b), we substituted the sequence of the 3′side of the stem with that of the 5′ side. Disruption of the stem by the substitution mutation affected RPE by more than twofold (*P* = 0.005; Fig. 5c) and propagation by twofold (*P* = 0.003; Fig. 5d). In a similar approach, the base pairing of the stem below the second bulge was disrupted by reciprocally substituting the sequences from both sides of the stem resulting in mutants SL2-7.1 and SL2-8.1 (Fig. 5b). In SL2-9A4, however, the disrupted base pairing in the stem of SL2-8.1 was restored in such a fashion that the sequences were exchanged between the two sides of the stem in the resulting *de novo* base paired stem (Fig. 5b). Base pairing destabilizing substitution mutations on either side of the stem in the case of SL2-7.1 and SL2-8.1 lowered the RPE by 1.5-fold and 1.9-fold, respectively, compared to the wild type (Fig. 5c); whereas propagation was reduced by 1.2-fold and 1.3-fold, respectively (Fig. 5d). In contrast, and quite unexpectedly, the SL2-9A4 mutant, in which compensatory mutation approach was supposed to restore base pairing actually showed drastic structural changes in Mfold predictions (data not shown). These structural changes were correlated with the reduced level of genomic RNA content and its propagation; lowering RPE by 2.7-fold (*P* = 0.0008) and propagation by twofold (*P* = 0.002) when compared to the wild type (Fig. 5c and d).

In an attempt to determine whether destabilizing the lower part of SL2 (just below the boxed area in Fig. 5a) would affect FIV RNA packaging, a series of mutations in this region were introduced. It was rather surprising that the majority of the mutations in this region of SL2 resulted in only a marginal reduction in packaging and propagation efficiencies (data not shown). This suggests that neither the primary sequence nor the RNA secondary structure of this region of SL2 is important for FIV RNA packaging. Such an assumption is consistent with our recent observation that SL2 is present in both genomic as well as spliced RNA and therefore is less likely to contribute towards packaging signal specificity.^31^

### Mutational analysis of *gag* sequences base pairing with sequences in R/U5 and UTR

**Fig. 6:**
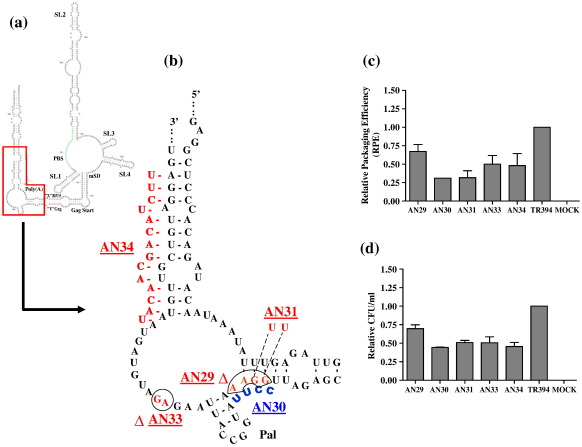
A representation of the deletion/substitution mutations introduced in the *gag* sequences base pairing with the 5′ end of the RNA genome in the predicted RNA secondary structure and their effect on transfer vector RNA packaging and propagation. (a) FIV RNA secondary structure highlighting the region showing the base pairing between the 5′ and 3′ ends of FIV RNA packaging determinants. (b) Enlarged view of the boxed area in Fig. 5a showing the specific mutations that were introduced. (c) RPEs of the transfer vectors containing deletion/substitution mutations in *gag*. (d) Relative transfer vector RNA propagation for each mutant expressed as Hyg^r^ CFU/ml of the viral supernatant and the data were derived after normalization to the transfection efficiency. The data are derived from at least three independent transfection and infection experiments except for the RPEs of AN30. Poly(A), polyadenylation sequence; PBS, primer-binding site; mSD, major splice donor; Pal, palindromic sequence.

It has been reported that packaging determinants of FIV consist of at least two discontinuous regions, one extends from R to approximately the first 150 nt of the 5′ UTR while the other is within the initial 100 nt of Gag.^19,20^^,^^22-24^ Consistent with these observations, we showed that the initial 105 nt of the RNA base pair with the sequences at the 3′ side of the mSD, including the first 100 nt of *gag* showing widespread LRIs (Fig. 6a).^31^ In order to ascertain whether this base pairing between the 5′ and the 3′ sides of the folded RNA is crucial for packaging at the structural level, we introduced a series of mutations in this region, as illustrated in Fig. 6b.

Testing these mutations using our *in vivo* packaging and transduction assay revealed that most of these mutations affected both RNA packaging (1.5–3.2-fold reduction in RPE) and propagation (1.4–2.3-fold reduction) when compared to wild type (Fig. 6c and d). This was despite the fact that the transfection efficiencies for the mutants and wild type clones were well within twofold for repeated experiments (data not shown). In mutant AN30, substitution of GGAA with CCUU should extend the palindromic stem (Fig. 6b) by allowing pairing of 5′ CUU with 3′ AAG (resulting pal 5′ CUUAAUGGCCAUUAAG 3′)and disrupt the stem 5′ of pal by preventing base pairing (Fig. 6b). Similarly, in the case of AN31, substitution of GG to UU should disrupt the base pairing and prevent the formation of the stem 5′ of pal (Fig. 6b). Interestingly, most severe and very comparable defects in RNA packaging were observed in these two mutants (more than threefold reduction in RPE when compared to the wild type; *P* = 0.01; Fig. 6c). As expected, AN30 and AN31 exhibited equally pronounced reduction in RNA propagation (2.3-fold and twofold reduction, respectively; *P* = 0.004 and 0.001; Fig. 6d). Surprisingly, deletion of GGAA in mutant AN29 (Fig. 6b), which was also targeted to disrupt the same stem (5′ of pal) showed only a modest (1.5-fold) decrease in both RNA packaging and propagation (Fig. 6c and d). Such a relatively minor effect of this mutant can be explained because the mutation introduced interferes only with low-affinity A-U and G-U base pairs, which might be relatively unstable.

The disruption of the run of six purines by deletion of AG in AN33 (Fig. 6b) resulted in a twofold drop both in RPE (*P* = 0.05; Fig. 6c) and RNA propagation (*P* = 0.008; Fig. 6d). Finally, in the mutant AN34, 14 nucleotides of Gag were substituted in an attempt to destabilize the stem 3′ of pal, which is formed by interactions with R (Fig. 6b). This mutant showed a more than twofold impairment in both RPE and RNA propagation (*P* = 0.0005; Fig. 6c and d). Taken together, the results presented in this section demonstrate that the majority of the mutations introduced in sequences involved in base pairing of the 5′ with the 3′ ends of FIV RNA significantly affected both transfer vector RNA packaging and propagation, which further authenticate the functional correlates of the predicted structure and the role of the identified structural elements towards FIV RNA packaging.

## Discussion

Using a combination of mutational and RNA structural analysis, the results presented here are clearly in line with the overall RNA secondary structure of FIV packaging determinants that we and others have proposed.^19,20^^,^^22^^–^^24^ The mutational analysis and computer predictions provide further evidence that the intramolecular interactions of these sequences at the structural level^31^ contribute crucially to the ability of FIV to recognize the genomic message from the plethora of cellular and spliced mRNAs.

One of the hallmarks of the FIV RNA secondary structure is the involvement of the 5′ and the 3′ end regions in extensive LRIs, including a heptanucleotide in R/U5 (5′ CCCUGUC 3′) that is complementary to a sequence (3′ GGGACAG 5′) found 342 bp downstream in the Matrix coding region of Gag, which is structurally conserved between all FIV isolates examined, including those that infect pumas, lions and Pallas cats.^31^ Similarly, in the case of HIV-1, it has been shown that a heptanucleotide sequence (5′ GCUUGCC 3′) downstream of the polyadenylation signal interacts with a complementary sequence (3′ CGAACGG 5′) located about 400 bp downstream in the Matrix coding region showing LRI.^34^ Disruption of either sequence by site-directed mutagenesis inhibited this LRI and suggested its pivotal role in regulating important steps in the retroviral life-cycle, including RNA packaging and dimerization.^34^ Additionally, even though there are considerable sequence dissimilarities among human and simian lentiviruses, the conservation of these interactions in all HIV-1, HIV-2, and SIV isolates further provides evidence supporting the functional significance of this LRI in the lentiviral life-cycle.^34^ Our mutational analysis directed towards ascertaining the functional significance of such an LRI in FIV showed that mutations designed to disrupt formation of this 7 bp helix diminished RNA packaging and propagation (Figs. 1b, 2c and 2d). This could be attributed to the RNA folding pattern rather than sequence of the LRI because reforming the LRI with heterologous complementary sequences in AN19 also led to restoration of RNA packaging and propagation (Fig. 3). This suggests strongly that such an interaction exist between the complementary heptanucleotides and that it is also imperative for crucial steps in the viral life-cycle. Our mutational analysis of the complementary heptanucleotides corroborates the LRI model with the observations made by Kemler *et al*.,^22^ who reported a threefold drop in RPE by deleting 13 nt of Gag, of which 3 nt are involved in the proposed LRI. Along the same lines, Browning *et al.*^19^ reported threefold reduction in FIV RNA packaging when no gag sequence was included in the FIV transfer vector.

The proposed RNA secondary structure and our mutational analysis data further suggest that the two core FIV packaging determinants (R/U5 + 150 bp UTR and 100 bp of *gag*) have complementary sequences that interact at the structural level to enhance packaging. The involvement of *gag* sequences as part of packaging determinants of retroviruses, where the core determinant seems to be upstream of the mSD (and hence part of all mRNAs), provide a convenient way of differentiating between spliced and unspliced mRNAs. This may be true for FIV as well as SIV and MPMV.^30,46^ *Gag* sequences, especially the first half of *gag*, might have a more mechanistic function. For example, HIV-2 has its major packaging determinants upstream of the mSD; thus, all HIV-2 mRNAs contain the packaging signal. HIV-2 has solved the problem of genomic mRNA recognition by using a unique sorting mechanism whereby primarily only those mRNAs capable of translating *gag in cis* can be packaged into the virus particle. These mRNAs are captured by the co-translated Gag polyproteins while being translated on polysomes in the cytoplasm.^11,47,48^ Although no direct evidence is presented here, it is possible that a similar co-translational mechanism might exist for FIV.

A characteristic feature of the retroviral life-cycle is the packaging of two copies of viral genomic RNAs in the form of a non-covalently linked RNA dimer. The conservation of this unique genome organization among retroviruses suggests strongly that a dimerized genome plays a vital role in the viral life-cycle.^49^ In retroviral replication, the process of RNA packaging is thought to be closely linked with RNA dimerization and, in some, the former depends on the latter. A DIS motif consisting of a characteristic 6 nt pal sequence for HIV-1^45,50^ has been found to be phylogenetically conserved in over 50 HIV-1, HIV-2 and SIV isolates.^49^ In addition, it was shown recently that a 10 bp pal sequence in the UTR, which is phylogenetically conserved in HIV-2 and macaque and sooty mangabey SIVs,^51^ is crucial for HIV-2 RNA packaging and dimerization.^38-40^ In the structure we published earlier, we identified a pal sequence (5′ AAUGGCCAUU 3′) in stem–loop 5, which is within *gag*. Semi-quantitative RT-PCR analysis of the mutants designed to test the significance of pal in FIV RNA packaging revealed that most of the mutations in pal reduced RNA packaging by 1.5 – threefold compared to the wild type (Fig. 4c), implicating, but not proving, a direct role of pal in RNA packaging. Such a reduction in RNA packaging and virus replication by pal mutants has been reported in HIV-2.^40^ Furthermore, deletions of *gag* sequences (including deletion of pal) in an FIV transfer vector have been shown to lower the packaging efficiency by more than threefold.^19,20^ Although we have observed the effects of pal mutations on RNA packaging, the effects of these mutations on RNA dimerization cannot be ruled out, which in turn may have negatively influenced FIV RNA encapsidation, especially if one argues that FIV RNA is packaged as a dimeric genome. Although a DIS (a prerequisite for dimerization) has not been firmly identified yet for FIV, the presence of a conserved prominent pal within the packaging signal region is suggestive. Therefore, it would be interesting to investigate the effect of pal mutations on RNA dimerization, which might differentiate the relative roles of pal in RNA dimerization and packaging. It was shown recently that the replication of HIV-1 containing mutations in DIS is largely producer cell-dependent and a DIS stem–loop is dispensable for HIV-1 replication in primary T-lymphocytes.^52-54^ Therefore, once ascertained that FIV pal in Gag functions as DIS, it would be interesting to investigate the effects of pal mutations in primary feline cells to determine whether FIV pal functions also or not in a cell-dependent fashion like that of HIV-1.

Our mutational analysis of SL2 showed only a modest reduction in RNA packaging and propagation efficiencies (Fig. 5). This might be explained by several possibilities. First, the deleted and/or substituted nucleotides both at the primary sequence as well as at the secondary RNA structural level might not be required for FIV RNA packaging. Alternatively, the entire SL2 might be needed as a whole for packaging and therefore more drastic mutations will need to be introduced into SL2 to appreciate effects on RNA packaging significantly. Second, other *cis*-acting elements might be independently promoting RNA packaging. For example, there is evidence that sequences in the 3′ end LTR also contribute, although minimally, to FIV RNA packaging^21^ and such sequences might have masked minor differences in packaging of SL2 mutants compared to the wild type. Third, although SL2 has been proposed to act as part of the packaging signal,^19,24^ it was shown recently to be part of both genomic and spliced RNA,^31^ suggesting its minimal role in providing specificity to the packaging signal, which is consistent with the modest reduction observed in our mutational analysis of SL2.

Alternatively, it could be that part of SL2 is involved in augmenting nuclear export of the viral genomic RNA. The packaging signal of some retroviruses, such as murine leukemia virus, and direct repeats of Rous sarcoma virus (which are involved in packaging) have been shown to play an important role in nuclear export of the viral genomic RNA and their further cytoplasmic transport towards the plasma membrane.^55,56^ It is therefore conceivable that some of these mutants are affecting the nuclear export of the unspliced RNA; however, because of the presence of MPMV constitutive transport element (CTE),^57^ we could not observe any transport defect exerted by SL2 mutants. This assertion stems from the fact that an internal loop in the ψ of HIV-1 has been found to closely resemble the HIV-1 Rev-responsive element and has been shown to bind the Rev protein,^43,58^ and mutations in this loop reduced nuclear export of viral genomic RNA.^59^ Given the fact that we have used a heterologous transport element, it is reasonable to assume that this might have influenced RNA packaging by indirect mechanisms, even though the CTE and Rev/RRE-mediated trafficking has been shown to have equivalent efficacy in other systems for getting RNA packaged.^42^ Nevertheless, to overcome this possible caveat and to see if there is any effect of these mutations on the nuclear export pathway (influencing RNA packaging), it will be important to study the effect of SL2 mutations in the absence of CTE as well as in the presence of homologous Rev/RRE regulatory pathway.

Using a biologically relevant assay, our mutational analysis and structural predictions in conjunction with the existence of functionally important LRIs in other lentiviruses provide additional proof for the presence of a similar widespread LRI between R/U5 and Gag in FIV as we proposed earlier, further providing important functional correlates with the published reports on FIV packaging signal. The study described here is a step forward towards our enhanced understanding of RNA structural elements in relation to RNA–RNA and RNA–protein interactions and should provide further insights about FIV RNA packaging and replication, which is imperative for the development of safe and efficient FIV vectors for human gene therapy.

## Materials and Methods

### Numbering system

Nucleotide designation for the FIV_Petaluma_ (34TF10) strain is based on GenBank accession number M25381.^60^

### Plasmid construction: FIV packaging construct, envelope expression plasmid and FIV transfer vectors

The FIV packaging construct MB22, which expresses FIV *gag/pol* genes from a human cytomegalovirus (hCMV)/intron A/promoter enhancer has been described and was used to package the transfer vector RNAs.^41^ The vesicular stomatitis virus glycoprotein (VSV-G)-based envelope expression plasmid (MD.G) has been described and was used to pseudotype the FIV particles generated from MB22.^61^

Substitution and deletion mutations introduced in different regions of the RNA secondary structure were cloned into the FIV-based transfer vector TR394 (Fig. 1a).^41^ Briefly, TR394 contains the entire 5′ UTR and 333 bp of Gag in addition to the *cis*-acting sequences needed for genome replication, including transcription, polyadenylation, encapsidation, reverse transcription and integration. TR394 contains a hygromycin resistance (*Hyg*^*r*^) marker gene expressed from an internal simian virus 40 (SV40) promoter (SV-Hyg^r^ cassette) facilitating the analysis of the effect of these mutations on FIV transfer vector RNA packaging and propagation. In TR394, we have replaced the U3 region of the 5′ end of FIV LTR with hCMV promoter to enhance the expression of our vectors in human cells. In addition, MPMV CTE has been inserted downstream of the SV-Hyg^r^ cassette in TR394 to facilitate the efficient nucleocytoplasmic transport and/or stability of transfer vector RNA (Fig. 1a).^57^

The desired mutations in different components of the proposed RNA secondary structure shown in Figs 1 and 4–6 were introduced using the splice overlap extension (SOE) PCR strategy.^62^ Such a strategy required two separate rounds of PCR. The first round consisted of two separate reactions (PCR 1 and PCR 2) using TR394 as a template and the primers used were designed in a fashion that will introduce the desired mutations. We used products from the above two PCRs as templates in the second round of PCR, and sense and anti-sense primers that were used in PCR 1 and PCR 2, respectively. The final PCR product, which contained the desired mutation(s), was digested with SpeI and BamHI sites and cloned into TR394 that was cleaved with the same restriction sites and the introduced mutations were confirmed by sequencing. The PCR amplifications were performed in 50 μl volumes using high-fidelity *Taq* polymerase in PCR super mix (Invitrogen) in the GeneAmp PCR system 9700 (Applied Biosystems).

The details of PCRs, primers, and the intermediate cloning steps are available from the authors upon request.

### Virus production and infection

Wild type or mutant transfer vectors, packaging construct MB22 and envelope expression plasmid MD.G were transfected into 293T cells in triplicate using calcium phosphate as described previously.^21,24^ pGL3 control DNA expressing firefly luciferase was added to the DNA cocktail to measure transfection efficiency as described earlier.^21,24^ The resulting supernatants from the transfected cultures were used to infect HeLa CD4^+^ cells and the infected cells were selected and stained for hygromycin-resistant (Hyg^r^) colonies as previously described.^41^

### Viral and cellular RNA isolation

To isolate the packaged viral RNA, viral supernatants containing virus particles were cleared of cellular debris via low-speed centrifugation (benchtop centrifuge, 4000 rpm for 10 min) followed by passage through a 0.2 μm cellulose acetate syringe filter and pelleted through a 20% (w/v) sucrose cushion by ultracentrifugation. The volume of supernatant to be used for pelleting virus particles for each mutant was determined following normalization with the transfection efficiency. The pelleted virus particles were resuspended in TNE buffer (50 mM Tris–HCl, pH 7.4, 100 mM NaCl, 1 mM EDTA, pH 8.0) and RNA was isolated using a TRIzol®-based method as described earlier.^21,63^

To fractionate the RNA into nuclear and cytoplasmic fractions, transfected cells were removed from the culture plates without trypsinization and then nuclear and cytoplasmic fractions were isolated as described previously.^21,63^

### Reverse transcriptase polymerase chain reaction (RT-PCR)

Before cDNA preparation, both viral and cytoplasmic RNA fractions were treated with DNase and amplified using vector specific primers OTR660 and OTR662 as previously described to ensure that the RNA preparations were devoid of any contaminating DNA.^24^ Next, cDNA preparations were amplified using the same vector-specific primers and the amplified products were used to calculate the relative packaging efficiency (described below). cDNA preparations were also amplified using β-actin-specific primers for both unspliced message (OTR582, sense, 5′ CCA GTG GCT TCC CCA GTG 3′; OTR581, antisense, 5′ GGC ATG GGG GAG GGC ATA CC 3′) as well as spliced message (OTR580, sense, 5′ TGA GCT GCG TGT GGC TCC 3′; OTR581, antisense, 5′ GGC ATG GGG GAG GGC ATA CC 3′). Amplification for unspliced β-actin mRNA was done in a multiplex PCR in the presence of primers/competimers for 18 S ribosomal RNA using quantum RNA classic II 18 S internal standard (Ambion).

### Relative packaging efficiency (RPE)

Viral and cytoplasmic cDNAs were amplified as described above using transfer vector-specific primers for calculating the RPE of each mutant. The PCR products were analyzed on agarose gels and transferred to a nylon membrane for Southern blot analysis as has been previously described.^21^ The blots were scanned using the Biometra gel documentation system and the absorbance of the various bands was quantified to calculate RPE for each mutant:

$$\left[\left(A_{\text{m}}\text{−}A_{\text{b}}\right)\text{/}\left(A_{\text{w}}\text{−}A_{\text{b}}\right)\right]\text{ /}\left[\left(A_{\text{cmN}}\text{−}A_{\text{b}}\right)\text{/}\left(A_{\text{cwN}}\text{−}A_{\text{b}}\right)\right]$$

where *A*_m_ is the absorbance of the mutant transfer vector RNA, *A*_b_ is the background absorbance, *A*_w_ is the absorbance of the wild type transfer vector RNA, *A*_cmN_ is the absorbance of the cellular mutant RNA normalized to relative transfection efficiency (RTE) and *A*_cwN_ is the absorbance of the wild type transfer vector cellular RNA normalized to RTE.

### Western blot analysis

As described earlier, one-third of the pelleted virus particles resuspended in TNE buffer were boiled for 5 min and then subjected to SDS-PAGE.^21^ Western blotting was done with 1:2500 dilution of polyclonal anti-serum from a cat infected with the Petaluma strain of FIV as described earlier.^21,41^ The blots were developed using the Super Signal West Pico Chemiluminescence Substrate (Pierce) and exposed to X-ray film.

### RNA secondary structure prediction and statistical analyses

Structural effects of the mutations were analyzed with Mfold sequence analysis software.^64,65^ The statistical analysis was done, using TR394 as the control, by one-way analysis of variance (ANOVA) with Dunnett's post test, using GraphPad Prism (version 5.01) for Windows (GraphPad software, San Diego, CA).
